# Uncertainty Evaluation of Two-Dimensional Horizontal Distributed Photometric Sensor Based on MCM for Illuminance Measurement Task

**DOI:** 10.3390/s25154648

**Published:** 2025-07-27

**Authors:** Jianguo Sun, Yueyao Wang, Yinbao Cheng, Guanghu Zhu, Jianwen Shao, Yuebing Sha

**Affiliations:** 1 College of Metrology Measurement and Instrument, China Jiliang University, Hangzhou 310018, China; s23020804051@cjlu.edu.cn (J.S.); p23020854098@cjlu.edu.cn (Y.W.); 2 Hangzhou Fangqian Technology Co., Ltd., Hangzhou 310030, China; zgh@fangqiantech.com; 3 Zhejiang Institute of Quality Sciences, Hangzhou 310018, China; shaybing@163.com

**Keywords:** distributed photometric sensor, Monte Carlo method, illuminance measurement, modeling of measurement uncertainty

## Abstract

In response to the demand for precise measurement of illuminance distribution in the quality control of LED monitoring fill light products and the iterative direction of secondary optical design, distributed photometric sensors have shown advantages, but their measurement uncertainty assessment faces challenges. This paper addresses the problem of uncertainty evaluation in photometric parameter measurement with a two-dimensional horizontal distributed photometric sensor and proposes an uncertainty evaluation framework for this task. We have established an uncertainty analysis model for the measurement system and provided two uncertainty synthesis methods, The Guide to the Expression of Uncertainty in Measurement and the Monte Carlo method. This study designed illuminance measurement experiments to validate the feasibility of the proposed uncertainty evaluation method. The results demonstrate that the actual probability distribution of the measurement data follows a trapezoidal distribution. Furthermore, the expanded uncertainty calculated using the GUM method was 21.1% higher than that obtained by the MCM. This work effectively addresses the uncertainty evaluation challenge for illuminance measurement tasks using a two-dimensional horizontal distributed photometric sensor. The findings offer valuable reference for the uncertainty assessment of other high-precision optical instruments and possess significant engineering value in enhancing the reliability of optical metrology systems.

## 1. Introduction

Industry 4.0 is centered around intelligence and data-driven approaches, accelerating the digital transformation process of the manufacturing industry [1]. As a precision optical measurement device, a two-dimensional horizontal distributed photometric sensor not only serves traditional optical parameter detection but also become the core link of industrial product quality control and the iterative direction of secondary optical design through precise measurement, which can improve product consistency and shorten research and development cycles [2]. Driven by the rapid advancement of semiconductor lighting technology, LED fill lights have gained widespread adoption in fields such as security monitoring, industrial inspection, and photography, owing to their advantages in high luminous efficiency, long service life, and low energy consumption. The two-dimensional horizontal distributed photometric sensor is primarily employed to measure key parameters of LED fill lights, including their illuminance distribution curve, spatial luminous intensity distribution, and effective emission angle. Engineers utilize this measurement data to conduct secondary optical design, aiming to optimize the lamp structure and enhance its illuminance performance [3,4].

Li [5] proposed a theoretical equation relating illuminance to image depth and achieved accurate measurement of LED fill light illuminance using binocular stereo vision depth measurement technology. Xu [6] developed a UAV-based road illuminance measurement system, significantly enhancing inspection efficiency. Through comparative analysis of LED road lamp illuminance using both on-site measurements and lighting software simulations, Medina [7] found that actual field measurements were consistently lower than the simulated values. This discrepancy was attributed to variations in lamp types and specific measurement locations. Mahlab [8] compared multiple methods for camera-assisted illuminance measurement. Omar [9] developed an loT-based solar illuminance measurement system and compared the performance of multiple sensors. Zhang [10] proposed a distributed measurement method that uses multi-point illuminance values for accurate work plane illuminance measurement. Gao [11] developed a semi-feedback closed-loop control scheme using PWM dimming that requires only sunlight contribution measurement. Their light-sensor prototype experiments validated the scheme’s efficacy in maintaining stable illuminance, offering a viable solution for energy-efficient urban lighting applications. Wang [12] proposed a zero-reference network grounded in the camera response model. The architecture extracts exposure ratio parameters from radiation images via a two-layer parameter generation module, then integrates these parameters with a brightness transformation function to accomplish single image low light enhancement. Menendez [13] proposed a design framework for indoor positioning–lighting collaborative systems incorporating task/surrounding area partitioning. By establishing empirical LED layout rules, the framework simultaneously satisfies horizontal illuminance requirements, uniformity metrics, and positioning system specifications, thus addressing the research gap in lighting characteristic analysis for composite positioning–lighting systems. Yan [14] proposed a computational ghost imaging calibration method using a white paper reference. This approach derives non-uniform illuminance distribution from a single all-white image and achieves dynamic correction of reconstructed images. Yoo [15] developed a binary low-light enhancement algorithm incorporating local brightness, contextual information, noise levels, and anti-saturation properties. This digital approach effectively eliminates artifacts induced by simplistic intensity amplification.

This article takes the two-dimensional horizontal distributed photometric sensor widely used in industrial lighting detection as the research object, studies the uncertainty evaluation problem of its illuminance measurement task, and provides a solution for the accuracy evaluation of similar complex instrument systems, demonstrating significant engineering application value.

## 2. Working Principle and System Composition

### 2.1. Working Principle

This optical measurement system combines optical, mechanical, and electronic technologies. We fixed the position of the photometric sensor and clamped the tested LED fixture at the mechanical reference origin of the fixture’s self-rotation axis and main axis, which are perpendicular to each other. We used a 532 nm wavelength laser pointer to achieve sub-millimeter coaxial alignment between the photosensitive surface of the photometric sensor and the optical center of the LED fill light. The horizontal rotation axes and vertical rotation axes of the two-dimensional turntable have a rotation range of −180°~180° and an angle accuracy of 0.1°, establishing a precise positioning platform for laboratory testing of spot illuminance uniformity.

**Figure 1:**
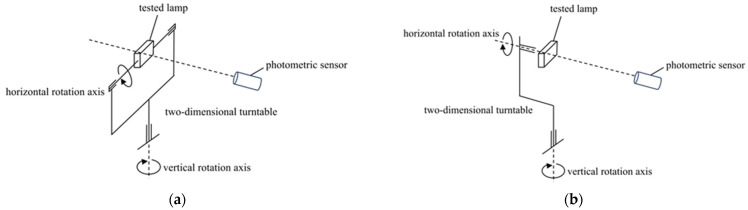
Measurement schemes. (**a**) Double column B-*β* measurement scheme; (**b**) single column C-*γ* measurement scheme.

According to the mechanical constraints, photometric characteristics, and testing standard requirements of the tested lamp, this system can be configured with a double column B-β measurement scheme or a single column C-γ measurement scheme through modular reconstruction, as shown in Figure 1.

**Figure 2:**
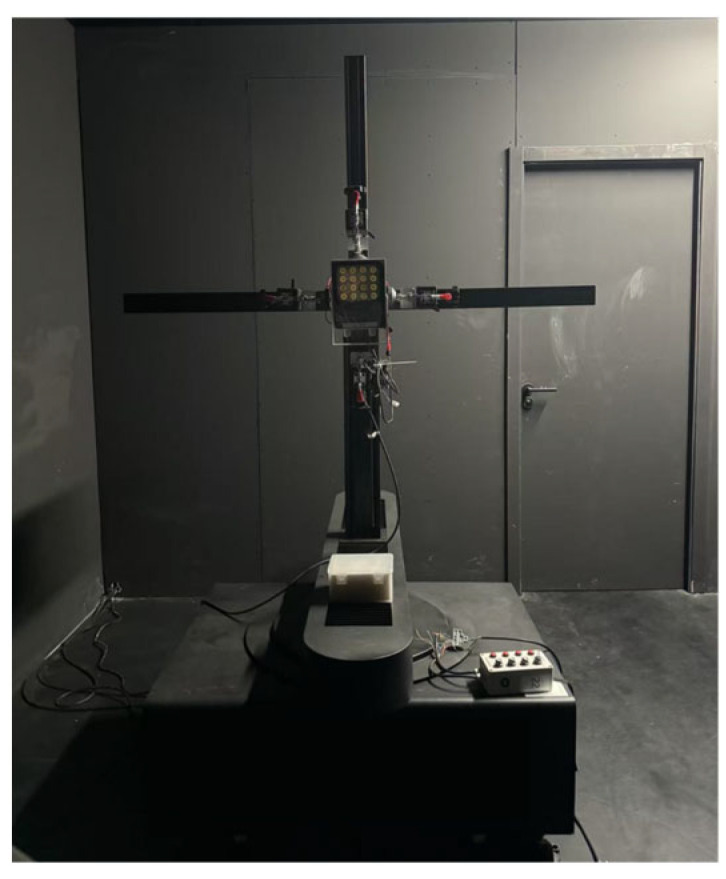
Experimental setup diagram.

The selection of measurement scheme mainly depends on the installation method and light intensity distribution characteristics of the tested lamp: if the tested lamp needs to dynamically adjust the pitch angle and change the direction of the optical axis in practical applications, one would select the double column B-β measurement scheme. If the tested light fixture is installed horizontally and with a focus on the uniformity of its horizontal illumination, the single column C-γ measurement scheme should be selected. Because the research object of this article is an LED monitoring fill light, the C-γ measurement scheme was selected, and its experimental setup diagram is shown in Figure 2.

### 2.2. System Composition

**Figure 3:**
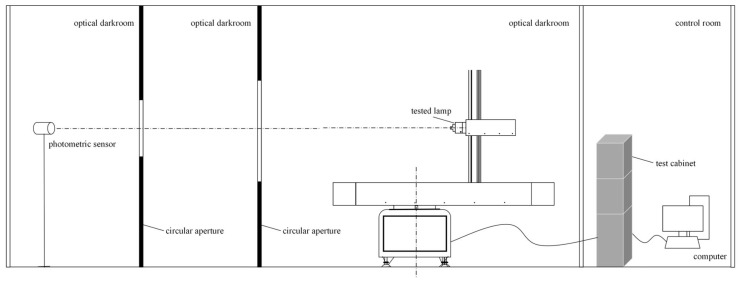
Controlled-environment photometric parameter measurement platform.

The system mainly consists of a photometric sensor, two circular apertures, the tested lamp, a two-dimensional turntable mechanism, a turntable controller, a cross-shaped positioning fixture, a testing cabinet, and a computer, as shown in Figure 3. The measurement environment is located in a professional optical darkroom, and its main structure is a black velvet cloth with a surface absorption rate of up to 0.99, which can effectively eliminate the interference of stray light. The darkroom is equipped with a temperature and humidity control system, which can accurately regulate the temperature and humidity of the measurement environment, ensuring the stability and repeatability of experimental conditions. The darkroom features an electromagnetic shielding function, which can effectively block external electromagnetic interference to ensure the accuracy of measurement data.

### 2.3. Deduction of Integral Formula for Illumination of Surface Light Source

The array LED fill light used for security monitoring is usually composed of multiple LED beads. By using lenses for secondary optical design, the discrete point light source group is transformed into an extended radiation field with surface-like light source characteristics, significantly improving the spatial consistency of the beam and the uniformity of the spot illumination. The Lambertian radiation characteristics formed through array integration and optical control enable the light source to exhibit a typical surface light source radiation distribution in engineering applications [16].

According to the basic formula of radiometry, the definition of brightness *L* is

(1) $$L=\frac{d\Phi }{d\Omega \cdot dscos\theta }$$

In the formula, dΦ is the radiated luminous flux propagated by the area ds of the element, dΩ is the solid angle element of the radiation direction of the light source, and dscosθ is the effective radiation area of a microelement.

**Figure 4:**
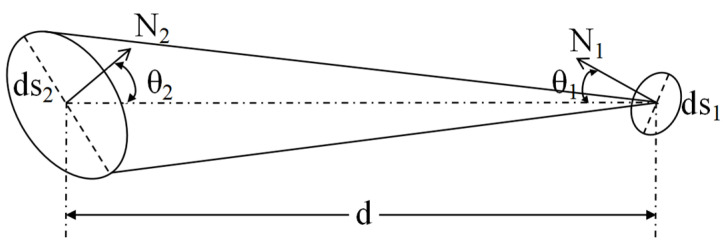
Radiation principle of surface light source.

During the radiation transfer process of a surface light source, a radiation transfer model is established by selecting the differential surface element ds_1_ on the radiation source surface S_1_ and the corresponding surface source ds_2_ on the irradiated surface S_2_, as shown in Figure 4. We assume that radiation source S_1_ has a uniform brightness distribution, where $\theta_{1}$ is the angle between the center line of the dihedral element and the normal to the surface of radiation source S_1_, and $\theta_{2}$ is the same. The luminous flux radiated by the area element ds_1_ into space is

(2) $$d\Phi_{1}=Ld\Omega_{1}\cdot ds_{1}cos\theta_{1},$$

(3) $$d\Omega_{1}=\frac{ds_{2}cos\theta_{2}}{d^{2}}.$$

From Formulas (2) and (3),

(4) $$d\Phi_{1}=\frac{Lds_{1}cos\theta_{1}ds_{2}cos\theta_{2}}{d^{2}}.$$

Therefore, the illuminance E_2_ of the radiation source area element ds_1_ on the irradiated surface is

(5) $$dE_{2}=\frac{d\Phi_{1}}{ds_{2}}=\frac{Lcos\theta_{1}cos\theta_{2}}{d^{2}}ds_{1}.$$

The above formula describes the differential illuminance dE_2_ generated by the area element ds_1_ of the radiation source surface S_1_ at the irradiated surface S_2_. Therefore, the illuminance generated by the radiation source surface S_1_ can be calculated through global integration, that is,

(6) $$E_{2}=\iint_{S_{1}}\frac{Lcos\theta_{1}cos\theta_{2}}{d^{2}}ds_{1}.$$

When the radiation source surface S_1_ is parallel to the irradiated surface S_2_ in space, the line connecting the centers of the two microelements is parallel to the normal direction of their respective surfaces, that is, the angle $\theta_{1}=\theta_{2}=0^{{}^{\circ}}$. Therefore,

(7) $$E_{2}=\frac{1}{d^{2}}\iint_{S_{1}}Lds_{1}=\frac{I}{d^{2}}$$

In the formula, *I* represents the luminous intensity of the radiant light source.

## 3. Uncertainty Evaluation Method

### 3.1. GUM Method

The standard uncertainty evaluation method serves as the core methodological foundation of the modern uncertainty theory system, directly determining the metrological validity of measurement results through its methodological framework [17]. The Guide to the Expression of Uncertainty in Measurement [18] and Monte Carlo method are two methodological paradigms in the evaluation of measurement uncertainty. The two have both differences and complementarity in theoretical basis, technical routes, and scope of application, together forming the technical system of measurement uncertainty evaluation methods.

The GUM method is based on statistical analysis and subjective probability distribution assumptions and is divided into Class A and Class B evaluations of standard uncertainty. Based on the measurement model $Y=f(X_{1},X_{2},\ldots ,X_{n})$, linearization is performed through Taylor series expansion, and the uncertainty propagation law is derived using the variance synthesis theorem:

(8) $$u_{c}\left(Y\right)=\sqrt{\sum_{i=1}^{N}{\left(\frac{\partial f}{\partial x_{i}}\right)}^{2}u{\left(x_{i}\right)}^{2}+2\sum_{1\leq i<j}^{N}\rho_{ij}\frac{\partial f}{\partial x_{i}}\frac{\partial f}{\partial x_{j}}u(x_{i})u(x_{j})}.$$

In the formula, $\frac{\partial f}{\partial x_{i}}$ is the sensitivity coefficient (1≤i<n), and $\rho_{ij}=\frac{u(x_{i},x_{j})}{u\left(x_{i}\right)u(x_{j})}$ is the correlation coefficient between the input variables $X_{i}$ and $X_{j}$(−$1\leq \rho_{ij}\leq 1$).

Under the conditions of simple linear models, the GUM method has become the “gold standard” for evaluating measurement uncertainty due to its high computational efficiency and reliable results. When applying the GUM method for uncertainty quantification analysis of complex measurement models, there are limitations: (1). For high-order nonlinear models, it is difficult to obtain sensitivity coefficients. (2). When the uncertainty components have correlation, the calculation of the correlation coefficient $\rho_{ij}$ is difficult and the correlation term cannot be ignored. (3) The GUM method is suitable for static measurement models and difficult to use to evaluate dynamic measurement uncertainty. (4). When there is a significant asymmetric distribution of uncertainty components, the synthesized standard uncertainty is inaccurate. (5). If the measurement model cannot be expressed explicitly, mathematical analysis and derivation are difficult to implement. (6). When the number of inputs is extremely large, the computational workload increases dramatically, making it difficult to calculate.

### 3.2. MCM Method

For complex measurement models, uncertainty cannot be accurately synthesized based on GUM. The MCM method [19,20,21,22,23] uses computer simulation technology to perform discrete random sampling on the probability density function of input variables. With the known probability density function distribution of each input variable, the actual distribution of output variables can be predicted. The implementation process is as follows: (1) Analyze the measurement principle and measurement system, determine the main sources of error in the measurement process, ignore minor error sources, and establish an uncertainty evaluation model $Y=f(X_{1},X_{2},\ldots ,X_{n})$ for the comprehensive measurement system. (2). Based on experience, determine the probability density function of each input quantity $X_{1},X_{2},\ldots ,X_{n}$, and infer the probability distribution of the output quantity through the propagation of the input quantity probability distribution. Common probability distributions include normal distribution, rectangular distribution, triangular distribution, and trapezoidal distribution. (3). Using Python language to randomly simulate sampling of various input quantities in the model, substitute the sampled input quantity $X_{i}$ into the model. The larger the simulation frequency *M* of the experimental sample size, the larger the sample size, and the closer the output quantity is to the real situation. Generally, *M* = 10^6^ is taken. The obtained *M* model values are sorted in strict increasing order, and the frequency distribution histogram of the output is drawn. The best estimated value *y* and its standard uncertainty of the measurement result are calculated based on the *M* model values.

(9) $$y=\frac{1}{M}\sum_{i=1}^{M}y_{i}$$

(10) $$u\left(y\right)=\sqrt{\frac{1}{M-1}\sum_{i=1}^{M}{\left(y_{i}-\bar{y}\right)}^{2}}$$

### 3.3. Uncertainty Analysis

**Figure 5:**
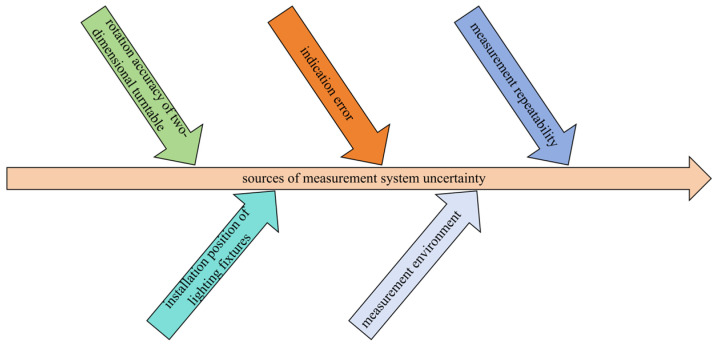
Sources of measurement system uncertainty.

The accuracy of measuring the illuminance of LED lamps using a two-dimensional horizontal distributed photometric sensor mainly depends on the collaborative cooperation of three systems: the mechanical motion system, photoelectric detection system, and environmental control system, which collectively determine the reliability of measurement results. In the process of dynamic measurement, the errors generated by each subsystem will gradually accumulate through the transfer function. The ultimate manifestation is the statistical distribution characteristics of the output data volume. According to ISO/IEC 17025:2017 [24], the sources of measurement uncertainty in this optical measurement system can be classified into three categories: inherent deviations of measuring equipment, technical proficiency of experimenters, and environmental interference, as shown in Figure 5.

The output quantity *Y* and various uncertain input quantities $X_{1},X_{2},\ldots ,X_{n}$ follow the mathematical model of $Y=f(X_{1},X_{2},\ldots ,X_{n})$. The sensitivity coefficient reveals its weight contribution to the output quantity *Y*. Due to the fact that the illuminance value of the lamp is directly measured by the optical measurement system, the uncertainty analysis model for the illuminance parameter measurement task is as follows:

(11) $$E=\bar{E}+\delta_{d}+\delta_{\theta }+\delta_{r}+\delta_{i}+\delta_{e}.$$

In the formula, *E* is the measurement result of the illuminance of the tested lamp, and $\bar{E}$ is the best estimate of illuminance.

## 4. Experimental Analysis

**Figure 6:**
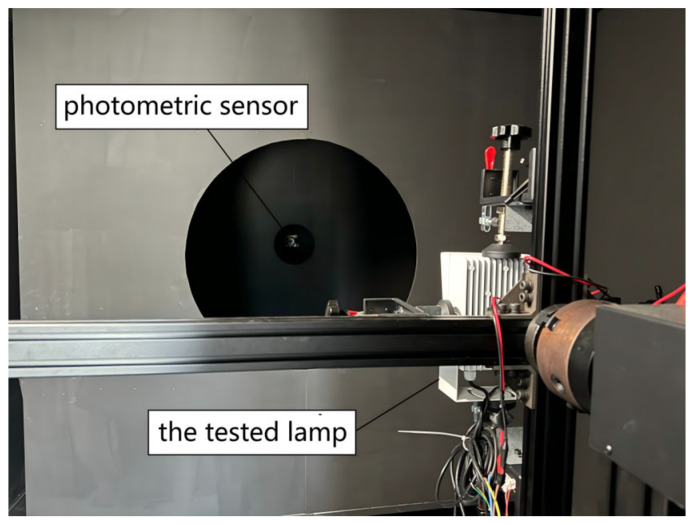
Illumination measurement.

The experiment used a two-dimensional horizontal distributed photometric sensor to measure the illuminance of a fill light fixture with an external size of (120×100×53) mm. The C-γ measurement scheme was selected. The brightness level of the lamp was (1–99) level. The testing distance was set to 10 m. Since the maximum size of the lamp was much smaller than the testing distance, it could be approximated as a point light source. The experimental environment was a uniform medium and a dark room without light leakage. Under rated voltage conditions, after 15 min of normal operation of the lamp, we visually inspected that the light spot in the supplementary lighting area remained stable, without obvious shaking. The light spot distribution was uniform, and there were no obvious dark areas. The illuminance measurement is shown in Figure 6.

The illuminance of the tested lamp was measured at low, medium, and high brightness levels. The results are shown in Table 1.

### 4.1. Uncertainty Evaluation of Measurement System

#### 4.1.1. Installation Position of Lighting Fixtures $\delta_{d}$

The geometric center of the luminous surface of the tested lamp is laterally or vertically offset from the center of the photometric sensor probe. The two centers are not aligned coaxially, causing optical axis mismatch and affecting the distribution of illumination. The ideal illuminance value can be calculated according to the inverse square law:

(12) $$E=\frac{I}{d^{2}}.$$

In the formula, *E* is the illuminance at the ideal installation position of the light source, *I* is the luminous intensity of the light source, and *d* is the distance between the light source and the photosensitive surface of the photometric sensor probe.

We used a handheld laser rangefinder to calibrate the distance *d* between the light source emitting surface of the tested lamp and the photometric sensor probe to 10 m. The ranging accuracy of the rangefinder is ±2 mm. Assuming it follows a uniform distribution, the uncertainty introduced by the accuracy of the laser rangefinder is

(13) $$u\left(d\right)=\frac{2\;mm}{\sqrt{3}}=1.15\;mm.$$

We calculated the partial derivative of *d* in Equation (12), and the standard uncertainty $u_{1}(E)$ of illuminance *E* is

(14) $$u_{1}\left(E\right)=\left|\frac{\partial E}{\partial d}\right|\cdot u\left(d\right)=\frac{2I}{d^{3}}\cdot u\left(d\right).$$

The relative uncertainty of illuminance is defined as the ratio of the standard uncertainty $u_{1}(E)$ to the measured value; then,

(15) $$\frac{u_{1}(E)}{E}=\frac{\frac{2I}{d^{3}}\cdot u(d)}{\frac{I}{d^{2}}}=2\cdot \frac{u(d)}{d}.$$

According to Table 1, the optimal estimate of illuminance at low, medium, and high brightness levels can be obtained. The uncertainty components at each brightness level are shown in Table 2.

According to Table 2, the uncertainty introduced by the installation position of the lighting fixtures at 99 brightness levels is

(16) $$u_{1}\left(E\right)=0.056\;lx.$$

#### 4.1.2. Rotation Accuracy of Two-Dimensional Turntable $\delta_{\theta }$

The angle accuracy of the two-dimensional turntable is 0.1°, which is 0.0017 rad. Therefore, the C-axis is 0.1°, and the γ-axis is 0.1°. The turntable controller allows only a single degree of freedom axis for angle adjustment at any time, following the non-cooperative action design of the horizontal and vertical rotation axes. Assuming it follows a uniform distribution, the uncertainty introduced by the turntable angle accuracy is

(17) $$u\left(\theta \right)=\frac{0.1^{{}^{\circ}}}{\sqrt{3}}=0.00100\;rad.$$

Due to the angle deviation of the turntable, the actual distance *d*’ from the tested lamp to the photometric sensor probe is

(18) $$d^{\prime }=\frac{d}{cos\theta }.$$

Currently, the illuminance value is

(19) $$E=\frac{I}{d^{2}}=\frac{I}{{\left(d^{\prime }cos\theta \right)}^{2}}.$$

We calculated the partial derivative of θ in Equation (19), and the standard uncertainty $u_{2}(E)$ of illuminance *E* is

(20) $$u_{2}\left(E\right)=\left|\frac{\partial E}{\partial \theta }\right|\cdot u\left(\theta \right)=\frac{2I}{d^{\prime 2}}tan\theta sec\theta^{2}.$$

The relative uncertainty is

(21) $$\frac{u_{2}(E)}{E}=2tan\theta \cdot u\left(\theta \right)=2\theta \cdot u(\theta ).$$

Selecting the best estimated illuminance value at a brightness level of 99, then,

(22) $$u_{2}\left(E\right)=0.00080\;lx.$$

Due to $u_{2}(E)$ being much smaller than $u_{1}(E)$, the influence of angle on the measurement results can be ignored.

#### 4.1.3. Measurement Repeatability $\delta_{r}$

The measurement repeatability $\delta_{r}$ reflects whether the measurement results are stable under the same experimental conditions. Ten consecutive measurements were taken on the illuminance of the lamp at low, medium, and high brightness levels. The best estimated value was the measurement average. The experimental standard deviation of a single measurement was calculated using the Bessel formula. Based on Table 1, 99-level brightness level data were selected, and the uncertainty introduced by repeatability is

(23) $$u_{3}\left(E\right)=\sqrt{\frac{\sum_{i=1}^{n}{\left(x_{i}-\bar{x}\right)}^{2}}{N(n-1)}}=0.026\;lx.$$

In the formula, $x_{i}$ represents the measured illuminance value, and *n* represents the number of measurements taken.

#### 4.1.4. Uncertainty Introduced by the Inaccurate Indication $\delta_{i}$

The inaccurate indication of the measurement system illuminance has been calibrated and issued a calibration certificate by the metrology department. The maximum inaccurate indication of illuminance at (0–300) lx is ±1.99 lx. Assuming it follows a uniform distribution, the uncertainty introduced by the inaccurate indication is

(24) $$u_{4}\left(E\right)=\frac{1.99}{\sqrt{3}}=1.15\;lx.$$

#### 4.1.5. Uncertainty Introduced by Measurement Environment $\delta_{e}$

The pressure in the darkroom is constant, and the temperature change does not exceed ±0.5 °C. During the experiment, the air flow velocity around the tested lamp is less than 0.2 m/s. A filter group is installed inside the photometric sensor probe to eliminate the influence of stray light in the darkroom. To verify the effect of stray light, all light sources in the darkroom are turned off, and the measured background illumination in the darkroom is 0.0272 lx. Assuming it follows a uniform distribution, the uncertainty $u_{5}(E)$ introduced by the measurement environment is

(25) $$u_{5}\left(E\right)=\frac{0.0272}{\sqrt{3}}=0.0157\;lx.$$

The uncertainty components of illuminance measurement are shown in Table 3.

According to the GUM guidelines, the composite standard uncertainty of the illuminance parameter measurement task is calculated using the variance synthesis theorem:

(26) $$u_{c}\left(E\right)=\sqrt{u_{1}{\left(E\right)}^{2}+u_{2}{\left(E\right)}^{2}+u_{3}{\left(E\right)}^{2}+u_{4}{\left(E\right)}^{2}+u_{5}{\left(E\right)}^{2}}=1.15\;lx.$$

When the probability of inclusion is *p* = 95%, *k* = 2, the expanded uncertainty is

(27) $$U=k\cdot u_{c}\left(E\right)=2.3\;lx.$$

The installation position of lighting fixtures $\delta_{d}$ is a uniform distribution, with a distribution range of [−0.056 lx, 0.056 lx]. The rotation accuracy of two-dimensional turntable $\delta_{\theta }$ is a uniform distribution, with a distribution range of [−0.00080 lx, 0.00080 lx]. The measurement repeatability $\delta_{r}$ follows a normal distribution with an expectation value of 0 and a standard deviation of 0.026 lx. The inaccurate indication $\delta_{i}$ is a uniform distribution, with a distribution range of [−1.15 lx, 1.15 lx]. The measurement environment $\delta_{e}$ is a uniform distribution, with a distribution range of [−0.0157 lx, 0.0157 lx].

**Figure 7:**
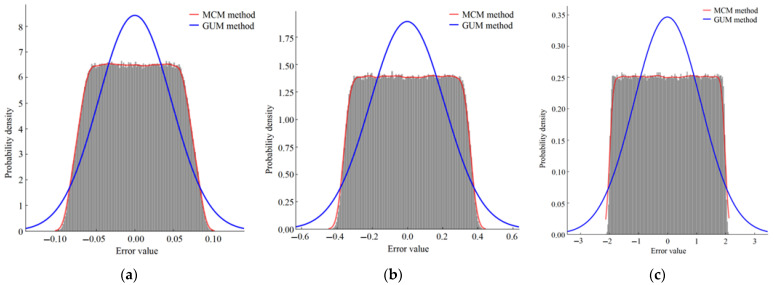
Error distribution histogram simulated using Python 3.12.4. (**a**) The brightness level is level 1; (**b**) the brightness level is level 50; (**c**) the brightness level is level 99.

Based on the Python development environment, 10^6^ computer simulation samples were taken for each input uncertainty component. The random sampling values of 10^6^ $\delta_{d}$*,* $\delta_{\theta }$, $\delta_{r}$, $\delta_{i}$, and $\delta_{e}$ loops were algebraically added together to obtain the error $∆E_{i}$ sample output values of 10^6^ illuminance measurements. The synthesized standard uncertainty is $u_{c}\left(E\right)=1.15\;lx$, which is consistent with the GUM method calculation results. We used Python 3.12.4 to draw a histogram of the distribution of $∆E=\delta_{d}+\delta_{\theta }+\delta_{r}+\delta_{i}+\delta_{e}$, arranged in ascending order from small to large, as shown in Figure 7c. When the confidence probability is *p* = 95%, the included interval is [−1.8978, 1.8841], and the extended uncertainty *U* = 1.90 lx, *k* = *U*/$u_{c}=$ 1.65. At the same time, the standard uncertainty $u_{c}$ is calculated to be 1.15 lx, corresponding to an inclusion probability of approximately 57.7%, which is lower than 68.27% estimated according to the normal distribution. The comparison of uncertainty results between the GUM and MCM calculations is shown in Table 4.

The uncertainty evaluation of illuminance measurement for the tested lamps with brightness levels of 1 and 50 follows the evaluation process for the 99-level brightness level mentioned above. The uncertainty distribution histograms are shown in Figure 7a,b.

## 5. Conclusions

This research focused on the uncertainty evaluation of a two-dimensional horizontal distributed photometric sensor for LED illumination measurement tasks. We established an uncertainty analysis model for the measurement system, analyzed the main sources of uncertainty, quantified each uncertainty component, and conducted a comparative analysis of uncertainty based on the GUM and MCM. After establishing the measurement model, MCM can efficiently achieve numerical simulation calculations. The standard uncertainty obtained by the two methods is basically the same. Experimental analysis reveals potential limitations of traditional uncertainty assessment methods. When the actual probability distribution of the output deviates from the assumed normal distribution of GUM, there is a systematic bias in the accuracy evaluation based on the assumption of normal distribution, resulting in a 21.1% increase in the extended uncertainty of traditional methods compared to the MCM results. This is because the main source of uncertainty in the measurement system is the indication error of the instrument, which is estimated according to a uniform distribution. Therefore, the uncertainty of the measurement results actually follows the trapezoidal distribution. The tail decay rate of the actual probability distribution is much faster than the expected normal distribution, resulting in an unreasonable *k* value selected based on the GUM method.

## Figures and Tables

**Table 1:** Illuminance measurement results at low, medium, and high brightness levels (unit: lx).

Sequence	Brightness Level		
	Level 1	Level 50	Level 99
1	0.651	132.28	243.32
2	0.633	132.29	243.43
3	0.632	132.35	243.27
4	0.659	132.26	243.49
5	0.622	132.34	243.38
6	0.643	132.37	243.26
7	0.639	132.26	243.35
8	0.652	132.29	243.40
9	0.623	132.38	243.29
10	0.629	132.24	243.47
average value	0.638	132.306	243.366
standard deviation	0.0127	0.050	0.082

**Table 2:** Uncertainty component of illuminance at low, medium, and high brightness levels (unit: lx).

Brightness Level	$\mathrm{Illumination}\;\mathrm{Value}\;\bar{E}$	$u_{1}(E)$	Expanded Uncertainty
level 1	0.638	0.000100	0.00020
level 50	132.306	0.030	0.061
level 99	243.366	0.056	0.112

**Table 3:** Uncertainty component estimation (brightness level: 99, unit: lx).

Uncertainty Component	Source	Expectation	Standard Deviation	Distribution
$u_{1}(E)$	$\delta_{d}$	0	0.056	uniform distribution
$u_{2}(E)$	$\delta_{\theta }$	0	0.00080	uniform distribution
$u_{3}(E)$	$\delta_{r}$	0	0.026	normal distribution
$u_{4}(E)$	$\delta_{i}$	0	1.15	uniform distribution
$u_{5}(E)$	$\delta_{e}$	0	0.0157	uniform distribution

**Table 4:** Comparison of GUM and MCM evaluation results (brightness level: 99).

Uncertainty Information	GUM Method	MCM Method
standard uncertainty $u_{c}$	1.15 lx	1.15 lx
	*p* = 68.27%	*p* = 57.70%
extended uncertainty *U* (*p* = 95%)	2.3 lx	1.90 lx
	*k* = 2	*k* = 1.65

## Data Availability

Data are contained within the article.

## Abbreviations

The following abbreviations are used in this manuscript:

GUM	Guide to the Expression of Uncertainty in Measurement
MCM	Monte Carlo Method
